# Algorithms and semantic infrastructure for mutation impact extraction and grounding

**DOI:** 10.1186/1471-2164-11-S4-S24

**Published:** 2010-12-02

**Authors:** Jonas B Laurila, Nona Naderi, René Witte, Alexandre Riazanov, Alexandre Kouznetsov, Christopher JO Baker

**Affiliations:** 1 Department of Computer Science & Applied Statistics, University of New Brunswick, Saint John, New Brunswick, E2L 4L5, Canada; 2 Department of Computer Science & Software Engineering, Concordia University, Montréal, Québec, H3G 1M8, Canada

## Abstract

**Background:**

Mutation impact extraction is a hitherto unaccomplished task in state of the art mutation extraction systems. Protein mutations and their impacts on protein properties are hidden in scientific literature, making them poorly accessible for protein engineers and inaccessible for phenotype-prediction systems that currently depend on manually curated genomic variation databases.

**Results:**

We present the first rule-based approach for the extraction of mutation impacts on protein properties, categorizing their directionality as positive, negative or neutral. Furthermore protein and mutation mentions are grounded to their respective UniProtKB IDs and selected protein properties, namely protein functions to concepts found in the Gene Ontology. The extracted entities are populated to an OWL-DL Mutation Impact ontology facilitating complex querying for mutation impacts using SPARQL. We illustrate retrieval of proteins and mutant sequences for a given direction of impact on specific protein properties. Moreover we provide programmatic access to the data through semantic web services using the SADI (Semantic Automated Discovery and Integration) framework.

**Conclusion:**

We address the problem of access to legacy mutation data in unstructured form through the creation of novel mutation impact extraction methods which are evaluated on a corpus of full-text articles on haloalkane dehalogenases, tagged by domain experts. Our approaches show state of the art levels of precision and recall for Mutation Grounding and respectable level of precision but lower recall for the task of Mutant-Impact relation extraction. The system is deployed using text mining and semantic web technologies with the goal of publishing to a broad spectrum of consumers.

## Introduction

Annotation of protein mutants with their new properties is crucial to the understanding of genetic mechanisms, biological processes and the complex diseases or phenotypes that may result. Despite attempts to manually organize variation information e.g. Protein Mutant Database [1] and Human Genome Variation Society [2], the amount of information is increasing exponentially so that such databases are perpetually out of date, and having a latency of many years. In recent years the extraction of mutation mentions from biomedical documents has been a growing area of research. A number of information systems target the extraction of mutation mentions from the biomedical literature to permit the reuse of knowledge about mutation impacts. These include work by Rebholz-Schuhmann et al. [3], *MuteXt* by [4] and *Mutation Miner* by [5]. The *MutationFinder* system [6] extended the rules of *MuteXt* for point mutation extraction. The *mSTRAP* system created by [7] is developed to extract mutations, represent them as instances of an ontology and use the *mSTRAPviz* client to query the populated ontology and visualize the mutations and annotations on protein structures / homology models. *Mutation GraB*[8] proposed the utilization of graph bigram to disambiguate the extracted protein point mutations. The *MuGeX* system extracts mutation-gene pairs [9]. Two recent systems by Krallinger et al. [10] and Winnenburg et al. [11] ground mutation mentions, as does the mSTRAP system [7].

However, little work exists on automatically detecting and extracting mutation impacts. An exception is EnzyMiner [12], which was developed with the aim of automatic classification of PubMed abstracts based on the impact of a protein level mutation on the stability and the activity of a given enzyme. In EnzyMiner, the predefined patterns of *MuGeX* are used to extract the mutations and a machine learning approach was taken to disambiguate the cell line names and strain names from mutations. Using a document classifier, the abstracts containing mutations without any impacts are removed and the remaining abstracts are classified into two groups of disease related and non-disease related documents, after which extracted mutations are listed for each group. In the case of the non-disease related abstracts, the documents are sub-classified into two groups: Documents containing impacts on stability; and documents containing impacts on functionality. This method for document classification can be useful in narrowing down search results but from the perspective of reuse and document annotation, more detailed methods for sentence-level detection, extraction and grounding of mutation impact information are required. In the current paper we present a rule-based approach for the extraction of mutation impacts on protein properties categorizing their directionality and grounding these entities to external resources. The system populates and RDF triple store and the algorithms are deployed as semantic web services.

### Content overview

The Methods section starts by describing our text mining pipeline (with named entity recognition and grounding of named entities to real-world entities), it continues to outline a mutation impact ontology specification and describes methods used to deploy mutation impact knowledge on the web. The Results section presents evaluations of the different subtasks and includes discussion of these results in the context of future improvements. Finally we provide a Conclusion and an outline of future work.

## Methods

### Named entity recognition

The first step of a mutation impact extraction system is to find named entities throughout the text, these include *mutations*, *protein properties* and words describing impact *directionality* as in the following sentence:

“The **W125F** mutant showed only a slight **reduction** of **activity** (V*_max_*) and a larger **increase** of **K***_m_* with 1,2-dibromoethane.” [13].

*protein-, gene-* and *organism names* also have to be recognized in order for the system to be able to properly ground mutations and protein properties:

“**Haloalkane dehalogenase (DhlA)** from **Xanthobacter autotrophicus GJI0** hydrolyses terminally chlorinated and brominated n-alkanes to the corresponding alcohols.” [14].

**Figure 1:**
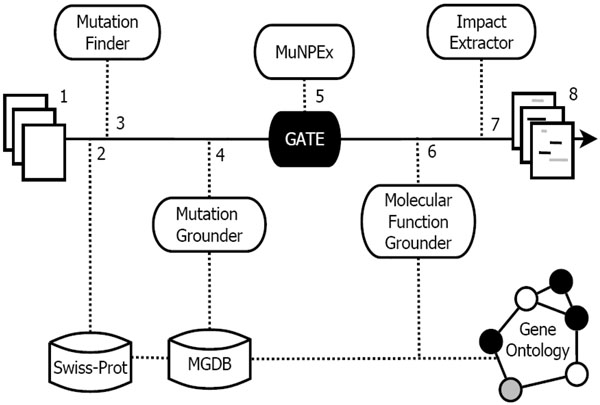
**Extraction and grounding framework**. Full-text documents (1) are run through a GATE pipeline with gazetteers derived from Swiss-Prot (2) and created with MutationFinder (3). Mutations and proteins are grounded (4). Protein properties are extracted with use of MuNPEx and custom JAPE rules (5) and grounded to the Gene Ontology when applicable. The impact extractor (6) makes use of the previous annotations to establish relations between mutants and impacts on protein properties. The output consists of annotated text (8).

We use GATE in combination with gazetteer lists created from a variety of resources and rules written in the JAPE language to find these entities. The following sections describe these methods in more detail. See Figure 1 for a system overview.

#### Mutations

To extract mutation mentions we used the MutationFinder system [6]. The system employs a complex set of regular expressions and is currently the best available tool for point mutation extraction. Full-text documents are first run through MutationFinder to create gazetteer lists containing mutation mentions that are compliant with the GATE framework. MutationFinder is also able to normalize mutations into *wNm* format, where *w* and m are one-letter codes for the wildtype and mutation residues, and *N* is the position on the amino acid sequence. Normalization is required prior to the mutation grounding task, we therefore add the normalized form as a feature to each gazetteer entry.

#### Proteins, genes and organisms

The protein database Swiss-Prot, a manually annotated part of UniProt KB [15], was used to select protein, gene and organism names. The use of Swiss-Prot is motivated by their high quality naming and mappings between names and protein sequences. The text format version of Swiss-Prot was encoded into our gazetteer lists compliant with GATE. Mappings between names and primary accession numbers and mappings between primary accession numbers and amino acid sequences are exported to a local database named Mutation Grounding Database (MGDB), for later use in the grounding / disambiguation step described in the *Grounding* section. Protein- and gene names containing more than one word are separated from names with only one word. The former are put in a gazetteer list for case insensitive matching of longer names to increase recall, and the latter are used for case sensitive matching of shorter names to increase precision. The organism names are put in a single gazetteer list for case insensitive matching containing both scientific (Latin genus and species) and English names.

#### Protein properties

Functions of proteins, as described in the Gene Ontology, are either activities e.g. *carbonate dehydratase activity*, or bindings to another entity e.g. *zinc ion binding.* To capture mentions of these functions in text we look for noun phrases with one of the words *activity*, *binding*, *affinity* or *specificity* as the head noun. This is accomplished by using *MuNPEx*, which is a multi-lingual noun phrase extraction component developed for the GATE architecture [16].

Kinetic variables are used to describe different features in an enzymatic reaction. They can for example describe how well the enzyme binds to the substrate or how efficient the overall catalysis is. Although they have to be interpreted in the context of the specific enzyme and substrates to be understood fully we still want to extract how these variables are impacted by mutations. This information can then be used in further enzyme dependent reasoning or by domain experts that are already capable of interpreting the meaning of these kinetic variables. In our implementation we annotate the Michaelis constant *K_M_*, the rate constant *k_cat_* and the compound variable *k_cat_/K_M_.* This is accomplished with rules written in the JAPE language which also makes sure variables are not part of a more complex variable or equation. Other protein properties such as *stability* are not considered in the current implementation.

#### Impact directions

To extract the actual impacts on protein properties we need terms describing directionality or the existence of a change. For example the negative impact on carbonate dehydratase activity of carbonic anhydrase II, which is due to two point mutations, might be described as: “*The double mutant had intact conformation but reduced catalytic activity (30-40%) compared to HCA II_pwt_*” [17]. In this example the word *reduced* and to some extent *intact* are keywords describing directionality of impacts.

In our implementation we used five different gazetteer lists categorized as positive, negative, neutral, non-neutral and negation. The gazetteers were created by domain experts who extracted words describing directionality from sentences containing protein functions. To escape the need for a stemmer, the gazetteers were extended with other grammatical forms of words already extracted. A total number of 337 sentences containing protein functions were extracted from a corpus containing documents about mutations on carbonic anhydrases and apolipoproteins and the resulting gazetteer lists contain a total of 85 words describing directionality. An overview of the direction gazetteer lists is presented in Table 1.

**Table 1:** Categorized directionality words.

Positive	Negative	(cont.)	Neutral	Negation	Non-Neutral
increase	abolish	loose	identical	without	affect
-increases	decrease	defect	similar	no	effect
-increased	reduce	disrupt	full	not	alter
-increasing	lower	diminish			differ
enhance	inhibit				
higher	impair				
improve					

### Grounding

Grounding is the task of cross-linking entities found in text with their real-world counterparts. In the case of proteins the entities, protein mentions are grounded when they have been assigned the correct UniProtKB ID, and for mutation entities, the grounding task is to map mutation mentions to the correct amino-acid residues of sequences stored in the UniProtKB [18]. In the case of protein functions we define grounding as establishment of a link from these mentions to the correct Gene Ontology concept. Kinetic variable grounding is straightforward in our current implementation, as we only consider three different variables; *K_M_, k_cat_* and *k_cat_/K_M_.* Links to the substrates being acted upon would serve as a more granular grounding and would increase the ability to query for impact information more precisely, but for the time being we do not establish these links to substrates.

#### Proteins and mutations

The method we use for protein and mutation grounding was previously described by [19] and is summarized below:

In the first stage a pool of candidate protein accession numbers is generated based on mappings of gene and protein names occurring in the target documents to accession numbers in MGDB. To ensure a comprehensive pool of candidate accession numbers, and avoid errors as a result of poor co-reference resolution techniques (i.e. not linking shorter names in text to the previously mentioned long form stated earlier in text), all accessions for names in MGDB with additional suffixes to the original protein or gene name are also extracted. A pool of candidate accession numbers is generated for each document and trimmed to contain only the most frequently occurring accession numbers. For these proteins all extracted organism mentions are cross checked. Accession numbers not related to any retrieved organism mentions are discarded and the protein sequences of candidate proteins are retrieved from MGDB.

In the second step mutations extracted from the text are mapped onto the candidate sequences using regular expressions generated from the mutation mentions extracted from the text. Mapping mentioned mutations to the correct position on the correct sequence is a non-trivial task. False positives can occur as a consequence of DNA level variations, plasmid names and cases where the numbering scheme used by authors can differ from the one used in sequence databases, e.g. as a consequence of N-terminal methionine cleavage or other post-translational modifications. These issues are discussed further in [19].

The mutation grounding algorithm briefly works as follows. For each possible pair of mutations, we create a regular expression by using the wildtype residues and the distance between them; for two normalized mutation mentions *w*_1_*N*_1_*m*_1_ and *w*_2_*N*_2_*m*_2_, sorted in the ascending order of *N_i_*, the regular expression will be *w*_1_ • {*N*_2_ – *N*_1_ – 1}*w*_2_. E.g. *A*378*C* and *S*381*L* will result in *A · ·S.* If a regular expression matches a sequence, we check for the remaining mutations in the set, one after another, taking into account the numbering displacement found when using the regular expression.

The output of the algorithm is the accession number and corresponding sequence onto which most mutations are grounded, which is considered to be the wildtype sequence of the protein described in the document. Mutation mentions that do not match the sequence are discarded and in cases where two sequences are identified, the sequence with least displacement from the mutation numbering in the paper is chosen.

#### Protein functions

For grounding of protein function mentions we use the Molecular Function part of the Gene Ontology as a reference vocabulary. The terms in the Gene Ontology are already used for annotation of Swiss-Prot entries to describe the properties of proteins. This means that we can leverage these mappings between the proteins we have grounded and protein functions we are looking for. We can then use the information on related functions to ground protein function mentions found throughout the document. In addition to creating links to Gene Ontology concepts the relevance of each protein function mention is scored based on its similarity to synonyms of a certain Gene Ontology concept. In order to measure this similarity the protein function mentions are first split into words, thereafter stop words are removed and finally the remaining words are stemmed using the Snowball English stemmer [20]. The resulting set of words (*N*) are then compared with each synonym (*G*) of the Gene Ontology concept, which are prepared in the same way, by measuring the relative intersection as below:

After comparisons have been made to all synonyms the highest similarity score is chosen and added as a feature together with the id of the related Gene Ontology concept to the protein function mention annotation. In the next section, *Relation detection*, we show how these similarity scores together with mutant-impact relation scores and impact scores are used to solve contradictions in the output annotations. In order to increase the number of synonyms and hence the number of highly and correctly scored protein function mentions, synonyms of ancestors to the retrieved Gene Ontology concepts are also used for comparison.

### Relation detection

In order to establish legitimate links between previously recognized and in some cases grounded entities, we need to detect the relations between them. For the purpose of mutation impact extraction we recognize relations between directionality words and protein properties which, taken together as a triple, constitute impact statements. Relations between mutants and these impacts are also detected. The two methods make use of heuristics based on entity distance.

#### Impacts

**Figure 2:**
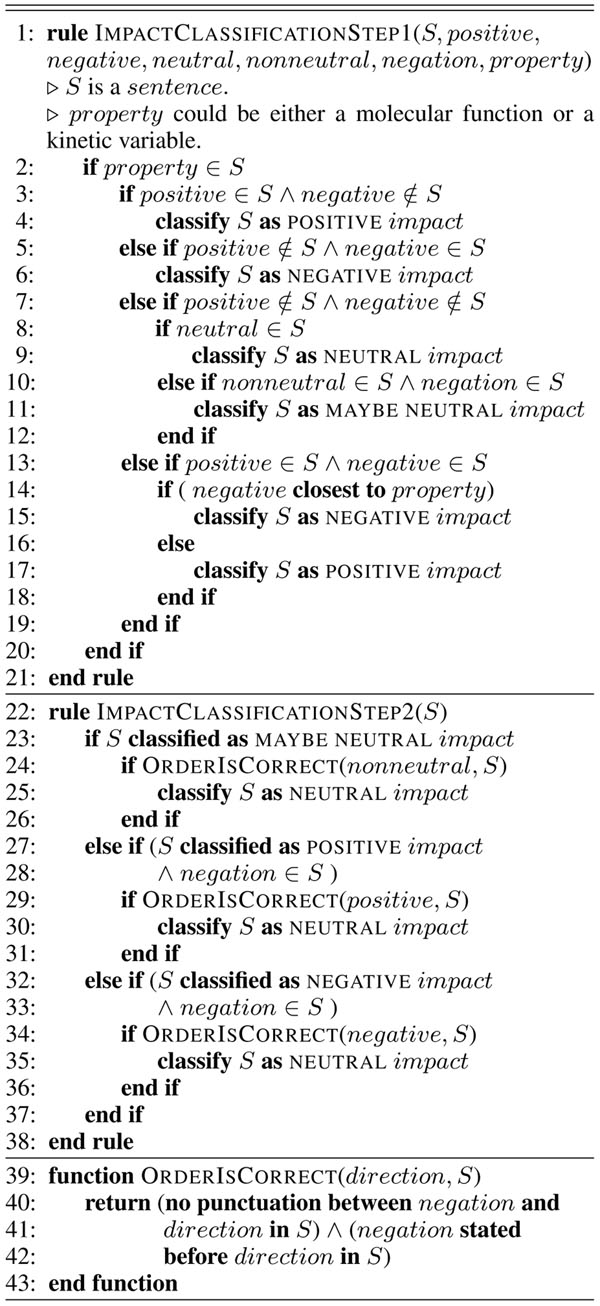
Rules for impact classification.

Impacts can be seen as relations between protein properties and words describing directionality, or change. In order to extract these relations we use a set of rules (Figure 2) which are applied to the documents with properties (protein functions and kinetic variables) and directionality words found in them.

Since impacts on different properties can occur in the same sentence; sentences containing two or more properties are split by looking for the comma character or the word *and.* If none of these delimiters are found the sentence is split just before or after the next or previous property, depending on order. The impacts are also scored according to the distance between directionality words and protein properties:

where *tokenDistance* is the number of space tokens between the directionality word and the protein property. If the directionality word would be a part of the noun phrase of a property the distance is set to 1.

#### Mutant-impact relations

When impacts have been extracted and correctly classified according to directionality, we need to find the mutant that has this change in protein property relative to the wildtype. Mutants can be described in many ways: (i) as a series of mutations e.g. “Arg172Lys+His65Ala”, (ii) with a short *nick name* specific for the paper e.g. “Mut1”, (iii) as a pronominal reference e.g. “The triple mutant” or (iv) simply by a single point mutation. In our implementation we say that each grounded mutation mention constitutes one single mutant. To extract the relation between mutants and impacts we say that when an impact is found, the closest mutants all have that impact. The closeness is measured by sentence distance and is scored as:

where *sentenceDistance* equals 1 if a mutation mention occurs in the same sentence as the impact and increases by 1 for each previous sentence, limited to at most three previous sentences. Only mutations with the shortest distance are considered.

To solve contradictions in the output annotations, e.g. when a mutant is said to have both negative and positive impact on a specific property the arithmetic mean of all scores gathered through the process are used, i.e. the mutant-impact relation score, the impact score and the similarity score between function mentions and Gene Ontology concepts. For kinetic variables the similarity score is omitted since it is not measured. A higher score means higher similarity to the Gene Ontology concept and shorter distance between directionality, property and mutant terms making the overall assertion more likely to be correct.

### Mutation impact ontology

**Figure 3:**
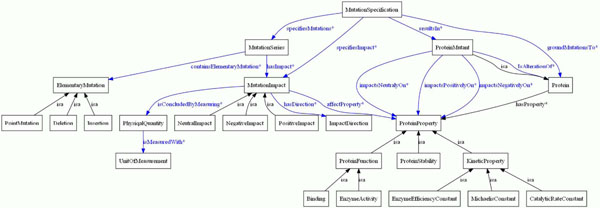
**Mutation impact ontology structure.** Visualization of top level concepts as *Mutation Specification, Protein, Mutation Impact* and *Protein Property* being connected through object properties. Detailed descriptions of the concepts are provided in Table 2.

In order to ensure the results of our text mining pipeline are reusable and understandable by both humans and machines we have formally specified the concepts used by our system in an OWL-DL ontology, with a small set of SWRL rules added for more convenient querying. The ontology we use is an extension of the ontology proposed by [21] and will serve as both the T-Box for a triple store populated with results from our text mining pipeline and for publishing our text mining pipeline as SADI services, making it possible to deploy our pipeline as semantic web services connected to other existing services. These two ways of publishing knowledge will be described in more detail in the next section, *Web based deployment.* Table 2 shows more precise definitions of the most important concepts and Figure 3 displays a schematic view of the concepts and the relations between them. In addition to object properties connecting instances of concepts, datatype properties are also used to associate data values with such instantances, e.g. *hasSequence* and *hasWildtypeResidue* associate string values with instances of *Protein* and *PointMutation* respectively. Some of the concepts are closely related to concepts in already existing ontologies. For example, the concept *ProteinFunction* in our ontology can be considered as equivalent to *Molecular Function* in the Gene Ontology. When making these alignments, it is possible to further enhance the querying ability and options for knowledge discovery. A user could, for example, search for all mutations that have positively impacted on a specific protein function, specified as a sub-concept of *MolecularFunction.* This type of query would not be possible without the grounding of protein properties, provided by our algorithm. The ontology, hereafter named Mutation Impact Ontology, is made publicly available [22].

**Table 2:** Concepts in the Mutation Impact Ontology and their descriptions.

Concept	Description
Protein	Proteins, also known as polypeptides, are organic compounds made of amino acids arranged in a linear chain and folded into a globular form.
Protein Mutant	A protein mutant is a protein where the amino acid sequence is altered compared to the wildtype protein. These alterations are called mutations.
Protein Property	The physical, chemical and biological properties of proteins. Stability and Function to mention a couple.
Elementary Mutation	An elementary change in the amino acid sequence of a protein.
Mutation Series	A set of elementary mutations.
Mutation Specification	An umbrella concept introduced as a link between mutations, their corresponding proteins, the impacts they cause and the texts.
Mutation Impact	A mutation impact describes a directional alteration of a protein.

### Web based deployment

**Figure 4:**
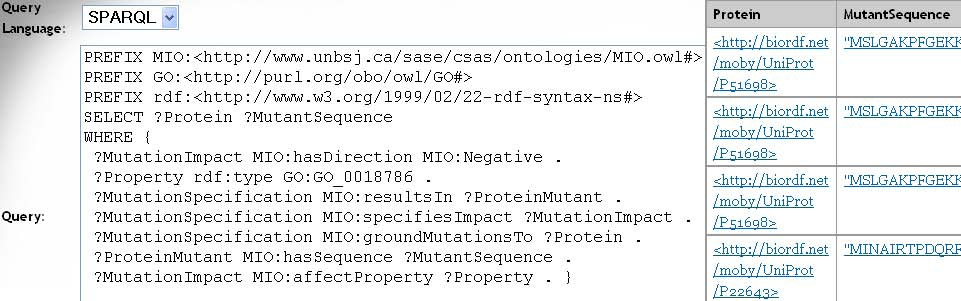
**SPARQL query and answers.** A SPARQL query expressing the natural language question “Which proteins have been mutated so that there is a negative impact on haloalkane dehalogenase activity and what are the sequences of the corresponding mutants?” is shown to the left. The first four answers (result rows) are displayed to the right.

**Figure 5:**
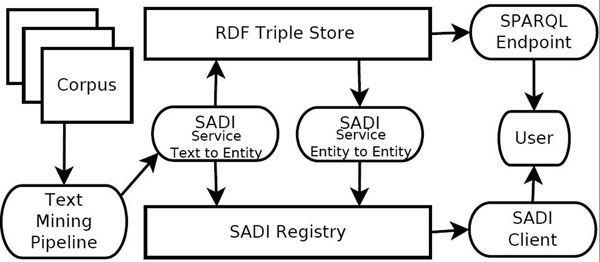
**Mutation impact knowledge flow.** The text-to-entity SADI service uses the text mining pipeline to extract mutations and impacts from a given text. The results are saved in an RDF triple store. The triple store can then be interrogated, either by a user through a SPARQL endpoint or by a second layer of entity-to-entity SADI services that in turn can be accessed through a SADI client.

The most straightforward way to deliver the results of our text mining pipeline to end users is to run the pipeline on available publications, store the results in a triplestore and provide a query interface. We have set up such a triplestore using Sesame [23], which is a framework that allows different storage and querying engines to be used via a unified interface. Our users can query the populated RDF triplestore via a SPARQL [24] endpoint [25]. Figure 4 shows an example query which, translated into a natural language question, reads “*Which proteins have been mutated so that there is a negative impact on haloalkane dehalogenase activity and what are the sequences of the corresponding mutants?*”. Figure 5 shows how mutation impact information is made available for the user through both SPARQL endpoints and SADI clients as discussed below.

#### SADI-compliant semantic web services

Although querying the triplestore can serve many useful information requests, such as searching for publications related to various biological entities, or just searching for links between the entities, we are aiming to make this data available in a format that is suitable for rapid data integration. This can be achieved by integrating our pipeline with other sources of semantically described biological data and analytical resources, so that queries can be made to our data combined with external data and data generated by externally hosted algorithms. For example, if some other resource is able to link proteins to pathways, combining it with our pipeline (that can link mutations to proteins) would make it possible to find a pathway in which a mutated protein participates. The SADI framework [26] provides a convenient way to facilitate such combinations. SADI is a set of conventions for creating Semantic Web Services (SWS) that can be *automatically discovered and orchestrated.* A SADI-compliant SWS consumes an RDF graph with some designated node (individual) as input. The output is an RDF graph similar to the input but with some new property assertions. The most important feature of SADI is that the predicates for these property assertions are fixed for each service. A declaration of these predicates, available online constitutes a *semantic description* of the service. For example, if a service is declared with the predicate *myontology*:*isTargetOf Drug* described in an ontology as a relation linking proteins to drugs, we know that we can use the service to search for drugs targeting a given protein. More importantly, such semantic descriptions allow *completely automatic discovery* and *composition* of SADI services (see, e.g., [27,28]). Practically, this means that the publication of our pipeline as SADI services will allow automatic integration with hundreds of external resources dealing with mutations, proteins and related biomedical entities, e.g., pathways and drugs. As an initial implementation with SADI, we created a service that takes a reference to a text, and outputs the property assertions derived from the input text, such as links to the identified grounded mutations. Note that those grounded mutations also have links to ungrounded mutations, proteins and impacts. This service can be mostly useful in combination with services that find documents, as well as for users just wishing to use our pipeline remotely (with no installation effort). In fact, we use this service ourselves to populate the previously mentioned RDF triple store. As the service output already constitutes an RDF graph no intermediate processing is necesssary.

We also created services that provide mappings in different directions: from entities to texts and from entities to entities derived from texts. In fact, all these services produce instances of *MutationSpecification*, which are blank nodes linked to other objects that may be of interest. For example, we can ask about grounded mutations applying to a certain protein, and the extracted *MutationSpecification* instances will lead us to relevant impacts, or just to the documents mentioning them. Our entity-to-text and entity-to-entity services serve data from the same triplestore providing the SPARQL interface. Our services are registered at the SADI Registry and can be viewed at [29].

#### Automatic data integration example

To exemplify SADI service composition, we present an example of a query which in natural language reads: “Retrieve all mutated proteins, together with their 3D-structure information and mutant sequence, where mutations had a positive impact on haloalkane dehalogenase activity.”

To answer this query, two services have to be used together. The first service is represented by the predicate *impactIsSpecifiedBy* (inverse for *specifiesImpact)* and, for a given mutation impact, retrieves a mutation specification containing protein and mutant information, which in part answers the service request. The second service is represented by the predicate *has3DStructure* from the central SADI ontology [30]. It makes use of the protein information retrieved by the first service to further retrieve the related 3D structure information in the form of Protein Data Bank identifiers.

The discovery and integration of these two services can be done automatically by the use of SHARE (Semantic Health and Research Environment) [28], a SPARQL query engine that enables composition of registered SADI services.

## Results

### Evaluation

To evaluate the methods of mutation grounding and impact extraction a gold standard corpus was built as an extension to the corpus used by [5] containing documents about haloalkane dehalogenases. Full-text papers mainly about a single haloalkane dehalogenase were chosen. They also had to contain more than one point mutation in order for our grounding algorithm to work properly. The resulting corpus contains 13 documents and a domain expert was able to extract 54 unique (per document) mutation mentions and 73 unique mutant-impact relations from the text of these documents, with tables and figures excluded. Mutants containing more than one point mutation were split so that each mutation was considered as one mutant, this was made to better evaluate the impact extraction task without interference from the variety of ways to describe mutants.

For both tasks we measure performance with precision and recall. In the case of mutation grounding precision is defined as the number of correctly grounded mutations over all grounded mutations and recall is defined as the number of correctly grounded mutations over all uniquely mentioned mutations. For mutant-impact relations precision is defined as the number of correct relations over all retrieved relations and recall is defined as the number of correct relations over all uniquely mentioned relations. In order for an extracted mutant-impact relation to be considered correct all the parts have to be correct i.e. the protein property that is being impacted, the direction of the impact and the causal mutation. The results are displayed in Table 3.

**Table 3:** Performance evaluation made on a haloalkane dehalogenase corpus

Task	Precision	Recall
Mutation grounding	0.83	0.73
Mutant-Impact relation extraction	0.86	0.34

## Discussion

The performance of the underlying algorithms for mutation grounding and mutation-impact detection show respectable levels of precision and recall. The performance of the grounding algorithm is in line with our previous evaluation on a medical corpus built from the COSMIC database [31] with an average *precision =* 0.84 and *recall =* 0.63. The lower performance of Mutant-Impact relations retrieval *(recall =* 0.34) in our current study is caused by several factors. Out of 45 *false negatives* (correct relations that were not retrieved) 16 were influenced by mutation mentions that were not grounded and 14 were caused by co-reference issues, e.g. when “*double mutant*” was used instead of mentions of single point mutations. Other contributing factors include shortcomings in our rules for extracting kinetic variables and protein functions which gave rise to 12 false negatives and lastly, our method for extracting directionality words which accounts for 8 false negatives. The two latter categories of false negatives can in some cases be illustrated by the special case when there is a total loss of function. This can be described in text as an inactive enzyme instead of a decrease of function relative to wildtype as in the below example sentences:

“Replacement of Trp-125 or Trp-175 with arginine leads to a **nonactive** enzyme.” [32].

“Mutation of Asp260 to asparagine resulted in a catalytically **inactive** D260N **mutant.**” [33].

We believe these issues can be addressed by developing methods for co-reference resolution of mutation mentions and by improving mutation extraction and grounding algorithms, as well as extending gazetteers containing words describing directionality. Textual descriptions of kinetic variables could also be used as an extension to our current abbreviation-centric method and therefore improve recall of Mutant-Impact relation extraction. Finally, the special cases where the impact is a total loss of function can be handled by a new set of rules connecting terms describing enzymes/mutants and terms describing inactivity. Until now the tools for the extraction of mutation mentions from text have been considered appropriate for augmenting the manual curation of mutation databases, providing candidate protein point-mutation impact suggestions [34], *de novo.* However the number of *reuse* cases where mutation information is used to facilitate new annotation and prediction algorithms is growing [7,11,35,36] albeit dependent on semi-automatic processing of information from databases or text mining pipelines.

The dedicated infrastructure we have developed for *fully automated* mutation impact extraction from unstructured text has a respectable level of precision of 0.86, albeit with moderate recall. Although further testing of these grounding and impact extraction algorithms on a larger corpus of documents from open access journals is required, using such platforms it will become possible to assess the range of impacts that have been investigated though mutational analysis of target protein sequences and the outcomes of these investigations. This will give researchers insight into the type and scale of improvements that have been made to enzymes using existing mutagenesis approaches. Moreover, cross referencing of these improvements with the methodologies used to generate the mutations will provide further guidance to scientists in deciding on strategies for further enzyme improvement, e.g. site directed mutagenesis versus directed evolution. Beyond the summarization of such information for trend analyses, extracted and grounded mutation impact annotations will also aid protein engineers when reviewing 3D visualizations of protein structures, as described by [7]. Finally the publishing of services delivering mutation impact information in a format that can be readily integrated with other services will facilitate the reuse of mutation impacts to other communities. e.g. as training data for Machine Learning algorithms [36], so that tools that predict the impacts of mutations can be improved.

## Conclusion

The challenges we addressed, namely extraction and publication of mutation impacts, required the development and deployment of advanced solutions leveraging named entity recognition, grounding techniques, knowledge representation for mutation impacts as well as the setup and registration of semantic web services. The major innovations were to: design novel impact grounding techniques and to couple this with existing approaches for mutation grounding to protein sequences; exploit the utility of the SADI framework to expose the grounding and relation detection algorithms as semantic web services. Once operational these services are readily findable and easy to integrate with existing semantic web services in the SADI registry. This combination provides enhanced access to legacy information using a contemporary publishing medium.

## Abbreviations used

GATE: General Architecture for Text Engineering; MuNPEx: Multi-lingual Noun Phrase Extractor; JAPE: Java Annotation Patterns Engine; MGDB: Mutation Grounding Database; OWL: Web Ontology Language; SWRL: Semantic Web Rule Language; SADI: Semantic Automated Discovery and Integration; RDF: Resource Description Framework; SPARQL: SPARQL Protocol and RDF Query Language; SWS: Semantic Web Service; SHARE: Semantic Health and Research Environment; COSMIC: Catalogue Of Somatic Mutations In Cancer;

## Competing interests

The authors declare that they have no competing interests.

## Authors' contributions

JBL developed the rules for grounding of mutations and protein properties, contributed to the ontology design and corpora annotation. NN contributed to the pipeline design and corpora preparation. RW participated in coordinating the work and contributed to the ontology design. AR developed the web based deployment and wrote the corresponding section. AK contributed to the methods for relation scoring. CJOB led the work coordination and study design. All authors contributed to the manuscript.

## References

[B1] Nishikawa K Ishino S Takenaka H Norioka N Hirai T Yao T Seto Y Constructing a protein mutant database Protein Eng 1993 7 5 733 10.1093/protein/7.5.733 8073043

[B2] Cotton RG Horaitis O The Challenge of Documenting Mutation Across the Genome: The Hu-man Genome Variation Society Approach Hum Mutat 2004 23 447 452 10.1002/humu.20038 15108276

[B3] Rebholz-Schuhmann D Marcel S Albert S Tolle R Casari G Kirsch H Automatic extraction of mutations from Medline and cross-validation with OMIM Nucleic Acids Res 2004 32 135 142 10.1093/nar/gkh162 14704350 PMC373272

[B4] Horn F Lau AL Cohen FE Automated extraction of mutation data from the literature: application of MuteXt to G protein-coupled receptors and nuclear hormone receptors Bioinformatics 2004 20 557 568 10.1093/bioinformatics/btg449 14990452

[B5] Baker CJO Witte R Mutation Mining-A Prospector's Tale Information Systems Frontiers 2006 8 47 57 10.1007/s10796-006-6103-2

[B6] Caporaso J Jr WB Randolph D Cohen K Hunter L MutationFinder: a high-performance system for extracting point mutation mentions from text Bioinformatics 2007 23 1862 1865 10.1093/bioinformatics/btm235 17495998 PMC2516306

[B7] Kanagasabai R Choo KH Ranganathan S Baker CJO A Workflow for Mutation Extraction and Structure Annotation J Bioinform Comput Biol 2007 5 6 1319 1337 10.1142/S0219720007003119 18172931

[B8] Lee LC Horn F Cohen FE Automatic Extraction of Protein Point Mutations Using a Graph Bigram Association PLoS Comput Biol 2007 3 2 e16 10.1371/journal.pcbi.0030016 17274683 PMC1794323

[B9] Erdogmus M Sezerman U Application of automatic mutation-gene pair extraction to diseases J Bioinform Comput Biol 2007 5 6 1261 75 10.1142/S021972000700317X 18172928

[B10] Krallinger M Izarzugaza JM Rodriguez-Penagos C Valencia A Extraction of human kinase mutations from literature, databases and genotyping studies BMC Bioinformatics 2009 10 Suppl 8 S1 10.1186/1471-2105-10-S8-S1 19758464 PMC2745582

[B11] Winnenburg R Plake C Shroeder M Improved mutation tagging with gene identifiers applied to membrane protein stability prediction BMC Bioinformatics 2009 10 Suppl 8 S3 10.1186/1471-2105-10-S8-S3 19758467 PMC2745585

[B12] Yeniterzi S Sezerman U EnzyMiner: automatic identification of protein level mutations and their impact on target enzymes from PubMed abstracts BMC Bioinformatics 2009 10 Suppl 8 S2 10.1186/1471-2105-10-S8-S2 19758466 PMC2745584

[B13] Kennes C Pries F Krooshof GH Bokma E Kingma J Janssen DB Replacement of tryptophan residues in haloalkane dehalogenase reduces halide binding and catalytic activity Eur J Biochem 1995 228 403 407 10.1111/j.1432-1033.1995.00403.x 7705355

[B14] Pries F Kingma J Janssen DB Activation of an Asp-124-Asn mutant of haloalkane dehalogenase by hydrolytic deamidation of asparagine FEBS Lett 1995 358 2 171 174 10.1016/0014-5793(94)01420-6 7828730

[B15] Boeckmann B Bairoch A Apweiler R Blatter M Estreicher A Gasteiger E Martin M Michoud K O'Donovan C Phan I Pilbout S Schneider M The Swiss-Prot Protein Knowledgebase and its supplement TrEMBL in 2003 Nucleic Acids Res 2003 31 365 370 10.1093/nar/gkg095 12520024 PMC165542

[B16] Multi-lingual Noun Phrase Extractor http://www.semanticsoftware.info/munpex

[B17] Svedhem S Enander K Karlsson M Sjbom H Liedberg B Lfs S Mrtensson LG Sjstrand SE Svensson S Carlsson U Lundstrm I Subtle Differences in Dissociation Rates of Interactions between Destabilized Human Carbonic Anhydrase II Mutants and Immobilized Benzenesul-fonamide Inhibitors Probed by a Surface Plasmon Resonance Biosensor Anal Biochem 2001 296 2 188 196 10.1006/abio.2001.5301 11554714

[B18] Witte R Baker CJO Towards a Systematic Evaluation of protein Mutation Extraction Systems J Bioinform Comput Biol 2007 5 6 1339 1359 10.1142/S0219720007003193 18172932

[B19] Laurila JB Kanagasabai R Baker CJO Algorithm for Grounding Mutation Mentions from Text to Protein Sequences Lecture Notes in Computer Science 2010 6254/2010 122 131 full_text

[B20] Snowball http://snowball.tartarus.org/index.php

[B21] Witte R Kappler T Baker CJO Enhanced semantic access to the protein engineering literature using ontologies populated by text mining Int J Bioinform Res Appl 2007 3 3 10.1504/IJBRA.2007.015009 18048198

[B22] Mutation Impact Ontology http://unbsj.biordf.net/ontologies/mutation-impact-ontology.owl

[B23] Broekstra J Kampman A van Harmelen F Sesame: A Generic Architecture for Storing and Querying RDF and RDF Schema The Semantic Web ISWC 2002 2002 54 68

[B24] SPARQL Query Language for RDF, W3C Recommendation 15 January 2008 http://www.w3.org/TR/rdf-sparql-query/

[B25] Mutation Impact RDF triplestore SPARQL endpoint http://unbsj.biordf.net/openrdf-workbench/repositories/mutation-impact-db/query

[B26] SADI framework http://sadiframework.org

[B27] Wilkinson MD Vandervalk BP McCarthy EL SADI Semantic Web Services - ’cause you can’t always GET what you want! APSCC 2009 13 18

[B28] Vandervalk BP McCarthy EL Wilkinson M SHARE: A Semantic Web Query Engine for Bioinformatics The Semantic Web (ISWC 2009) 2009 367 369

[B29] Registered SADI Services http://unbsj.biordf.net/mutation-impact

[B30] Central SADI Ontology http://sadiframework.org/ontologies/predicates.owl

[B31] Forbes S Bhamra G Bamford S Dawson E Kok C Clements J Menzies A Teague J Futreal P Stratton M The Catalogue of Somatic Mutations in Cancer (COSMIC) Curr Protoc Hum Genet 2008 57 10.11.1 10.11.26 10.1002/0471142905.hg1011s57 PMC2705836 18428421

[B32] Lau EY Kahn K Bash PA Bruice TC The importance of reactant positioning in enzyme catalysis: A hybrid quantum mechanicsymolecular mechanics study of a haloalkane dehalogenase Proc Natl Acad Sci USA 2000 97 9937 42 10.1073/pnas.97.18.9937 10963662 PMC27632

[B33] Krooshof GH Kwant EM Damborsky J Koca J Janssen DB Repositioning the Catalytic Triad Aspartic Acid of Haloalkane Dehalogenase: Effects on Stability, Kinetics, and Structure Biochemistry 1997 36 9571 9580 10.1021/bi971014t 9236003

[B34] Caporaso JG Deshpande N Fink JL Bourne PE Cohen KB Hunter L Intrinsic evaluation of text mining tools may not predict performance on realistic tasks Pac Symp Biocomput 2008 13 640 651 PMC2517250 18229722

[B35] Bauher-Mehren A Furlong LI Rautschka M Sanz F From SNPs to pathways: integration of functional effect of sequence variations on models of cell signalling pathways BMC Bioinformatics 2009 10 Suppl 8 S6 10.1186/1471-2105-10-S8-S6 PMC2745588 19758470

[B36] Bromberg Y Rost B SNAP: predict effect of non-synonymous polymorphisms on function Nucleic Acids Res 2007 3823 3835 10.1093/nar/gkm238 17526529 PMC1920242

